# Grit and perceived teacher support associations with Chinese language achievement: the mediating role of emotion in Thai high school Chinese classrooms

**DOI:** 10.3389/fpsyg.2025.1573713

**Published:** 2025-05-26

**Authors:** Li Pan, Xinyi Li, Xianyue He, Huiqin Luo, Qizhen Gu

**Affiliations:** ^1^School of Humanities, Arts and Education, Shandong Xiehe University, Jinan, China; ^2^KIS International School Reignwood Park, Bangkok, Thailand; ^3^Graduate School of Business and Advanced Technology Management, Assumption University, Bangkok, Thailand

**Keywords:** anxiety, boredom, Chinese language learning, enjoyment, grit, perceived teacher support

## Abstract

**Introduction:**

Chinese language learning is gaining considerable attention from learners worldwide, leading many countries, including Thailand, to make it a mandatory subject in primary and secondary education. However, many Chinese learners lack interest in learning Chinese, resulting in inadequate Chinese language proficiency. Chinese language learners’ grit and emotional commitment to Chinese language learning are key factors in determining their language learning success, and the support of Chinese as a Foreign Language teachers is also crucial in this process. Thus, this cross-sectional study investigates the associations of students’ grit and perceived teacher support with their Chinese language achievement while considering the potential mediating role of emotions (enjoyment, anxiety, and boredom).

**Methods:**

The study included 665 high school students from two public and three private schools in Bangkok. A questionnaire and an HSK exam paper were the instruments used in this study. Partial least squares structural equation modeling was applied to analyze the data.

**Results:**

The findings indicated that perceived teacher support, grit, and three emotional factors were each significantly associated with Chinese language achievement. While all three emotions mediated the relationship between perceived teacher support and Chinese language achievement, only foreign language learning enjoyment exerted a mediating role in the association between perseverance and Chinese language achievement.

**Discussion:**

With insights for Chinese language educators and educational administrators, this paper highlights the importance of fostering learning perseverance, supportive learning environments, and meeting students’ emotional needs to improve Chinese language learning achievement.

## 1 Introduction

In recent years, the number of Chinese as a foreign language (CFL) learners has increased by 20% per year, and there have been more than 25 million CFL learners worldwide (Zhou, 2023). Many countries have officially incorporated Chinese language courses into school education, such as Australia, New Zealand, Singapore, and Thailand (Duangmanee and Waluyo, 2023). Thailand has the largest number of CFL learners among less-developed countries (Xu et al., 2022). Owing to Thailand’s close economic, cultural, and political connections with China, a pressing demand exists for qualified professionals proficient in Mandarin and Thai to foster comprehensive cooperation between the two nations (Xu et al., 2022). In collaboration with China, the Royal Thai Government has established 16 Confucius Institutes, underscoring its commitment to advancing Chinese language education and fostering cultural exchanges between the two nations (Cao and Tananuraksakul, 2023). The Thai Ministry of Education has listed Chinese as a highly recognized foreign language and is dedicated to its promotion throughout all educational levels in Thailand (Ewe and Min, 2021).

Chinese language learning is mandatory in many Thai schools for students’ K12 education, with most Thai students beginning their structured Chinese education as early as primary school grade one (Duangmanee and Waluyo, 2023). According to Ning and Tananuraksakul (2024), over 2,000 Thai primary and secondary schools offer Chinese language courses, with student enrollment exceeding 1 million. For Thai high school students, Chinese has become a critical component of university entrance exams and a key determinant for accessing higher education opportunities (Zhang and Chinokul, 2023). Since 1998, the Chinese language has been officially integrated into Thailand’s high school curriculum, becoming a foreign subject in Thai university entrance exams (Ewe and Min, 2021). A considerable number of Thai high school students, especially those with relatively lower English proficiency, often invest more effort in learning Chinese, thereby gaining a competitive advantage in the Thai university entrance examinations (Zhang and Chinokul, 2023).

HSK (Hanyu Shuiping Kaoshi—Chinese Proficiency Test) is one of the most widespread and largest standardized examinations to test the Chinese language proficiency of non-native speakers of Chinese, which has been flourishing worldwide and is an important part of the development of globalization of Chinese language learning (Sun, 2023). The HSK is a standardized instrument for evaluating the language competency of CFL learners, effectively reflecting their proficiency in listening, speaking, reading, and writing in Chinese (Liu, 2022). The Chinese government annually invites Thai students with an HSK level 4 or above to study at leading universities in China, offering full scholarship support, which is undeniably a significant attraction for many Thai high school students (Peng et al., 2021). As a result of this policy, the number of Thai overseas students enrolled in Chinese universities has escalated from 667 in 2000 to 28,608 in 2018, with consistent year-on-year growth in recent years (Liang and Li, 2023).

Foreign language learning is a process in which students must interact dynamically with different people, among which interaction with teachers is particularly crucial (Liu and Li, 2023; Ma et al., 2022). Perceived Teacher Support (PTS) refers to learners’ subjective perceptions of the various kinds of support provided by teachers in the language learning process. Support from teachers can create a safe and friendly foreign language learning environment for language learners and guide them in generating positive learning emotions, which in turn effectively enhances their language proficiency (Sadoughi and Hejazi, 2022). Most Thai CFL teachers are Chinese, and some of them usually encounter problems such as insufficient language teaching experience, lack of understanding of the local culture, and difficulties in Thai language communication (Duangmanee and Waluyo, 2023; Ewe and Min, 2021). As a result of these problems, many Thai students feel that the support and assistance provided by their CFL teachers is insufficient, dampening their enthusiasm for learning Chinese and hindering the development of their Chinese proficiency (Duangmanee and Waluyo, 2024). Therefore, perceived teacher support (PTS) was included in this study as one of the factors affecting Thai students’ Chinese language achievement (CLA).

In language learning, grit (GR) is learners’ sustained passion for and perseverance toward long-term language goals (Teimouri et al., 2022). GR is characterized by steadfast commitment that enables language learners to overcome setbacks and maintain progress despite encountering setbacks or plateaus (Heydarnejad et al., 2022). Chinese is an extremely challenging language for non-native Chinese speakers, and only those who exhibit unwavering perseverance in their learning can master it through continuous practice and accumulation (Chai and Bao, 2023). Thai students must exert considerable effort to overcome the language barrier while learning Chinese, owing to the phonetic tonal differences, the memorization requirements of intricate character structures and stroke rules, and the grammatical disparities between Chinese and Thai (Ning and Tananuraksakul, 2024). Even at the university level, the Chinese proficiency of many Thai students remains low, with one of the most significant reasons being their loss of grit (GR) in Chinese learning (Cao and Tananuraksakul, 2023). Therefore, GR is also considered a critical factor in this study.

Emotions are indispensable in language learning (Dornyei and Ryan, 2015). Li and Wei (2023) Indicated that three emotions, foreign language enjoyment (FLE), foreign language anxiety (FLA), and foreign language boredom (FLB), significantly influence language learners’ language achievement, particularly FLE, which has great potential to enhance language proficiency. FLE is defined as a positive emotion directly associated with learning activities, denoting learners’ active involvement and favorable experiences with the language itself. As a Buddhist-majority nation, Thai students are exposed to Buddhist cultural concepts from an early age, which profoundly shapes the emotional aspects of their learning processes (Yuenyong and Yuenyong, 2012). The Buddhist cultural concept of “*sukha*” (true happiness and well-being)—referring to the state of happiness achieved through the elimination of suffering and the pursuit of inner peace and social harmony—profoundly influences Thai education, thus shaping students’ perception that learning should be an enjoyable and pleasurable experience (Dellios, 2025). Influenced by the cultural concept of “sukha,” Thai students attach significant importance to FLE in language learning, as they posit that language acquisition should inherently be a pleasurable process (Yang and Chanyoo, 2022). Cao and Tananuraksakul (2023) indicated that Thai students are more inclined toward learning Chinese due to their enjoyment of the Chinese classroom atmosphere. Enhancing the FLE is posited to foster sustained motivational engagement among Thai students and concurrently contribute to improving language learning outcomes (Toomnan, 2024). As such, FLE was included in this study as a positive emotion.

FLA refers to “a distinctive complex of self-perceptions, beliefs, feelings, and behaviors related to classroom language learning arising from the uniqueness of the language learning process” (Horwitz et al., 1986). FLA is a predominant negative emotion in foreign language learning and a crucial determinant of learning outcomes and classroom performance (Shen, 2021). It has remained the most extensively discussed emotional variable in foreign language learning research (Liu et al., 2025). Sukjairungwattana (2023) pointed out that some Thai students have negative emotions due to the monotonous learning environment and difficulties in the Chinese language, which leads to their loss of interest and motivation in Chinese learning. Thai students often experience FLA when studying Chinese, which is closely linked to the significant challenges posed by the Chinese language and their language learning environment (Ning and Tananuraksakul, 2024). However, in Thai Buddhist cultural contexts, students often attribute academic challenges, such as difficulties in learning Chinese, to the “*kamma*” (the law of cause and effect), interpreting these struggles as consequences of past-life misdeeds, which can foster self-perceptions of inherent unsuitability for learning and reducing their anxiety (Dhammasami, 2018). Furthermore, the Thai cultural concept of “*sati*” (mindfulness) refers to the focused observation of present-moment physical and mental states, through which Thai students, starting from childhood, are trained in Buddhist meditation practices to cultivate the ability to maintain equanimity and thereby better manage stress (Fry, 2018). Based on Xu et al. (2022), the Buddhist culture in Thailand, together with its emphasis on pleasure and relaxation, can shape Thai students’ perceptions of anxiety and their ways of coping with anxiety, thus impacting their Chinese learning achievement (CLA).

Boredom is defined as the aversive experience of having an unfulfilled desire to be engaged in a satisfying activity (Fahlman et al., 2013), and it has garnered significant attention in foreign language studies in recent years (Li et al., 2023). FLB is also one of the common negative emotions among Thai students in Chinese language learning. The Buddhist concept of “*Metta*” (loving-kindness) denotes a cultivated mental state of benevolence, compassion, and empathy for everyone cultivated through a systematic meditation practice regularly undertaken by Thai students (Tongsupachok et al., 2024). Profoundly influenced by “Metta,” Thai students tend to remain reticent rather than articulate constructive feedback to their teachers in tedious classrooms, driven by their cultural emphasis on deference to authority and avoidance of interpersonal conflict (Nakamura et al., 2021; Tongsupachok et al., 2024). However, this well-intentioned benevolence results in learning contents and tasks assigned by teachers failing to align with students’ expectations, preferences, and needs, thereby leading to the gradual accumulation of FLB (Nakamura et al., 2021). Factors contributing to FLB in the Chinese language classroom include unidimensional curriculum design and teaching strategies, excessive complexity of Chinese language content, and insufficient Chinese communication opportunities (Chen et al., 2024). Nakamura et al. (2021) posited that FLB could lead to numerous negative consequences on Thai students’ language learning, such as impeding their learning engagement, diminishing their learning interest, and eventually causing a decline in their language achievement. It is also necessary to study the influences of the two negative emotions, FLA and FLB, on Thai students’ Chinese language learning within the specific context of Thailand.

Research on foreign language learning is usually more focused on English as a Foreign Language (EFL), while research on CFL remains insufficient (Liu and Rao, 2024). While Chinese language education has gained significant traction in Thailand with a substantial learner population (Xu et al., 2022), existing research primarily focuses on investigating the current situation and recommending pedagogical techniques (Cao and Tananuraksakul, 2023; Duangmanee and Waluyo, 2023), with limited empirical exploration of the relationship between language achievement and psychological factors. This study investigates the association of students’ GR and PTS with their CLA in Thai high school Chinese language classrooms, considering the mediating role of emotional factors (FLE, FLA, and FLB).

## 2 Literature review

### 2.1 Perceived teacher support (PTS)

Self-determination theory (SDT) and the social support model provide contrasting definitions of teacher support (TS). Self-determination theory defines TS as the teacher’s responsibility to promote students’ intrinsic motivation and learning engagement by satisfying their autonomy, competence, and relatedness needs (Deci and Ryan, 2000). Autonomy pertains to learners’ inclination for choices and making decisions in their learning progress; competence refers to their desire to feel effective in accomplishing tasks; and relatedness underscores the significance of feeling connected and supported inside the educational setting (Deci and Ryan, 2000). In the social support model, TS is defined as a multidimensional construct comprising four components: informational support, instrumental support, emotional support, and appraisal support, which teachers systematically provide to enhance students’ academic and personal development (Tardy, 1985). Informational support refers to the provision of domain-specific advice; instrumental support refers to the allocation of tangible resources such as financial assistance and time; emotional support involves the demonstration of affection, trust, and empathy; and appraisal support is the delivery of timely feedback on student performance (Malecki and Elliott, 1999).

Self-determination theory emphasizes psychological needs for motivating autonomy, competence, and relatedness, whereas the social support model directs more detailed attention to the specific forms of support teachers offer students in real-world instructional contexts (Deci and Ryan, 2000; Malecki and Demaray, 2002). Thai CFL learners, especially non-Chinese major students, often engage passively in Chinese language learning, and their interactions with Chinese teachers are mostly confined to the classroom (Srisinthon, 2024). As a result, Thai students particularly value the instructional resources and information teachers provide (Kaur et al., 2015). This makes the social support model especially relevant for capturing the specific types of support observed in actual teaching contexts. “*Kreng Jai*” (deferential harmony), a deeply rooted Thai cultural norm, reflects the tendency to restrain personal desires or opinions out of respect for others and a desire to preserve social harmony (Areemit et al., 2021). Influenced by “Kreng Jai,” Thai students consider teachers as authorities and paternal figures. They perceive behaviors that exhibit individual autonomy, such as independently selecting learning content, as challenging teachers’ authority and potentially harmful to the teacher-student relationship. This perspective regards teachers as ‘knowledge providers’, resulting in Thai students seeing TS just as practical assistance, such as instructional feedback and resource provision (Kaur et al., 2015). Therefore, this study adopts the social support model’s definition of TS, defining PTS as Thai students’ perceptions of their Chinese teachers’ emotional, instrumental, informational, and appraisal support throughout their Chinese learning. Self-determination theory posits that teachers’ emotional and cognitive support can fulfill students’ needs, thereby enhancing students’ emotional experiences in learning and promoting the improvement of academic performance (Zhang and Hu, 2025). According to Cohen and Wills (1985), teacher support fosters a safe, encouraging environment that increases positive emotions, decreases negative emotions, and accelerates learning progress (Huang et al., 2024; Zhao and Yang, 2022).

While such findings are compelling, they mainly originate from EFL research contexts. Hejazi and Sadoughi (2023) discovered that TS might significantly decrease FLA and enhance English competence among Iranian EFL learners. Nonetheless, their findings may not be entirely applicable to the CFL context in Thailand, where students often display passivity driven by “Kreng Jai.” Tao et al. (2022) meta-analysis showed that PTS was positively and significantly associated with language learning achievement, especially for high school students facing pressure from higher education pursuits or employment prospects. Although this study incorporated data from a wide range of foreign language learning contexts, it still focused mainly on EFL scenarios. Given CFL’s unique challenges in phonological systems, Chinese character memorization, and other domains, the applicability of existing conclusions to Thai CFL classrooms requires further validation.

Piechurska-Kuciel (2011) indicated a significant negative correlation between PTS and FLA among Polish secondary school EFL learners. Liu et al. (2023) study of Chinese high school EFL learners found that PTS was negatively and significantly associated with FLA. Zhao and Yang (2022) found that PTS was significantly and positively associated with FLE and negatively with FLB among Chinese high school EFL learners. Wu et al. (2023) examined Chinese university EFL learners and discovered that PTS positively correlated with FLE and was significantly and negatively associated with FLB. However, these aforementioned studies were all conducted in EFL contexts and culturally distinct educational settings (European and Chinese), and it is important to note that learners in these studies typically engaged in voluntary English learning. Conversely, although Thai students need to study Chinese from an early age, they often engage in a more passive learning approach owing to the design of the Chinese curriculum and the impact of Thai culture. These findings, though insightful, may not be directly transferable to the Thai CFL context, where cultural and curricular factors differ considerably. Consequently, an empirical study is required to re-investigate the association of PTS with language learning emotions within Thai CFL contexts.

Research on the impact of PTS on learners’ language learning emotions and achievement in CFL settings is still relatively scarce and needs further empirical evidence. Du et al. (2017) found that TS promotes positive emotions in Danish beginner CFL learners, thereby increasing their motivation and CLA. Instead of using a scale to measure the TS, Du et al.'s (2017) study examined various teacher identities, teaching styles, and attitudes to gauge their relationship with Chinese learning. Bao et al. (2021) found that the PTS of Greek CFL learners effectively promoted trust between teachers and students, greatly increasing learners’ confidence and motivation, which in turn met their emotional needs. This study employed a single-case research design involving interviews with a CFL teacher participating in the “Sino-Greece Online Chinese Language Classrooms” project, which differs from the present empirical study based on scales and models. Zhou (2023) study indicates that when CFL teachers provide support such as effective error correction, positive interaction, and timely guidance, it can significantly enhance the motivation and willingness to communicate. These findings were based on a qualitative study of six Scottish CFL learners and did not explore the relationship between teacher support and CLA. Quantitative research exploring the relationship between PTS and CLA in CFL contexts remains underexplored, and this study aims to address this gap in the literature.

Most previous studies have focused on European learners, whose motivation, cultural values, and language environments significantly differ from those of Thai high school students. Learning Chinese poses significant challenges for most Thai CFL learners, whether because of the distinct tonal pronunciation and norms compared to the Thai language or the complexity of writing and memorizing Chinese characters, which can be considerably more difficult than the alphabet (Ning and Tananuraksakul, 2024). The concept of “Kreng Jai” impacts the hierarchical relationships between teachers and students, leading to distinctive perceptions of PTS among Thai language learners in contrast to those from other cultural backgrounds (Areemit et al., 2021). For example, Thai students may perceive the teacher’s strictness as supporting or caring rather than pressure. Exploring the association of Thai CFL learners’ PTS with their foreign language learning emotion and CLA could benefit CFL teachers in understanding the crucial role of PTS in cross-cultural language instruction, hence enhancing instructional support techniques to elevate students’ learning experiences and outcomes. Based on previous research findings, this study proposes the following hypotheses.

*H1*: PTS is positively associated with CLA.

*H1a*: PTS is negatively associated with FLA.

*H1b*: PTS is negatively associated with FLB.

*H1c*: PTS is positively associated with FLE.

### 2.2 Grit (GR)

As a psychological trait, GR is influenced by the positive psychological turn and has gained prominence in applied linguistics research (Dewaele and Dewaele, 2017; Teimouri et al., 2022). GR is defined as perseverance and passion for long-term goals, comprising two core components: maintaining enthusiasm and interest in tasks and persistence toward long-term goals (Duckworth et al., 2007). GR has become an essential predictor of success in a variety of fields and has also been emphasized and explored by numerous L2 researchers (Wang, 2021). As a positive personality trait, GR in L2 encompasses persistence of effort (POE) and consistency of interest (COI) (Teimouri et al., 2022). GR is a crucial non-cognitive factor in language learning that not only forecasts learners’ language proficiency but also addresses numerous long-term challenges by maintaining learners’ interest and persistent effort (Alamer, 2021). GR in language learning is not a fixed trait, but a malleable mental construct, meaning learners can cultivate and reinforce it through intentional effort and strategic engagement (Khajavy et al., 2021; Tang et al., 2019). GR fosters persistent motivation among foreign language learners despite challenges, hence improving language proficiency (Paradowski and Jelińska, 2024). GR, an essential construct in positive psychology that emphasizes individual strengths over weaknesses, is a notable non-cognitive predictor of academic achievement (Vela et al., 2015). Drawing on broaden-and-build theory (Zhao and Wang, 2023a) stated that GR, as a positive personality attribute, influences language acquisition by affecting emotions, hence improving learners’ engagement and performance. According to the Conservation of Resources Theory (COR), resources are categorized into four types: personal resources, social resources, material resources, and energy resources (Hobfoll, 2011). GR in language learning is conceptualized as a personal resource comprising two core dimensions: POE and COI (Liu et al., 2025). These two dimensions are regarded as personal psychological resources that enable learners to manage pressure, maintain motivation, and thus enhance academic performance (Halbesleben et al., 2014).

Teimouri et al. (2022) investigated Persian native EFL learners and found that GR positively correlated with English learning achievement. Fathi and Hejazi (2024) discovered that GR was significantly and positively associated with English language proficiency among Iranian EFL learners. Both studies were carried out in EFL contexts and did not include the mediating role of emotions on the relationship between GR and language achievement. Moreover, influenced by “sati” (mindfulness), the development of GR and its impact on the language competency of Thai students may vary from the previous research. Thai students are skilled at improving their GR for language learning through Buddhist practices such as meditation. Considerable research has been undertaken to examine the impact of GR on emotions related to language acquisition. Khajavy and Aghaee (2024) found that GR was significantly and positively associated with FLE and negatively with FLA among Iranian EFL learners. Zhao and Wang (2023a) found that Chinese EFL learners’ GR was positively and significantly associated with FLE and negatively with FLB. Similarly, Bensalem et al. (2024) discovered that the GR of EFL university students in Saudi Arabia was significantly associated with their FLE and FLB. The aforementioned research did not concurrently examine the association of GR with the three emotions (FLA, FLB, and FLE) and language performance within a single model. Under the dual influences of the formidable challenges of Chinese language learning and Buddhist culture, the interacting dynamics relationship among Thai CFL learners’ GR and language learning emotions has not been comprehensively examined via an empirical study. However, such exploration is of great significance for revealing the interconnected relationship among culture, emotion, psychological traits, and language learning.

GR-related research in the CFL context remains in its infancy. Sun et al. (2024) discovered that the GR of young CFL learners in New Zealand was positively and significantly associated with their Chinese speaking proficiency. It is important to note that Sun et al.’s (2024) CLA may not exactly match the study’s findings because it is based on the self-assessed oral performance of CFL learners rather than standardized tests. Zhao et al. (2024) demonstrated that GR in Arab CFL learners was significantly associated with FLE but not FLA, with FLE as a mediator between GR and CLA. The findings of Zhao et al.’s (2024) study, which focused on online Chinese language instruction during the COVID-19 pandemic, may not be applicable to regular offline Chinese language instruction as well. Zhao and Wang (2024) found that perseverance of effort in GR significantly affected FLA and FLE but not FLB among CFL learners in mainland China, while consistency of interest in GR did not significantly affect any of these three emotions. All of the participants in Zhao and Wang’s (2024) study were CFL students studying in China, all of whom had a high level of Chinese language competency and rather better Chinese language learning environment than CFL students studying Chinese in other countries.

While previous studies have examined the impact of GR on language learning emotions (FLA, FLB, and FLE) and CLA within the context of CFL, there is a lack of relevant research about GR for Thai learners. The GR of Thai language learners is characterized by sustained perseverance, pleasure of the learning experience, proficient anxiety regulation, and distinct goal orientation, all of which are intricately connected to Thailand’s Buddhist cultural heritage (Freiermuth et al., 2021). Having been immersed in Buddhist culture from childhood, many Thai students extend the self-discipline fostered by Buddhist rituals, such as daily chanting, to their language learning, believing that hard work in the present will pay off in the future (Freiermuth et al., 2021). Thus, the researchers intended to illustrate the unique mechanisms of GR on emotions and language achievement in the context of Buddhist culture and to provide empirical evidence for cross-cultural Chinese language teaching. The following hypotheses were proposed in this study.

*H2*: GR is positively associated with CLA.

*H2a*: GR is negatively associated with FLA.

*H2b*: GR is negatively associated with FLB.

*H2c*: GR is positively associated with FLE.

### 2.3 Emotions

Foreign language learning emotions refer to the various emotional states and psychological reactions experienced by learners in the process of foreign language learning (Plonsky et al., 2022). Control-value theory (CVT) provides theoretical support for research on the impact of emotions in the learning process (Pekrun, 2000). According to Pekrun and Stephens (2010), CVT categorized emotions into activating emotions (e.g., enjoyment, pleasure, and anxiety) and deactivating emotions (e.g., boredom, disappointment, and relaxation). Pekrun and Stephens (2010) further argued that positive emotions can enhance learners’ attention as well as increase motivation and engagement, thereby improving learning achievement, while negative emotions may increase the consumption of cognitive resources, weaken motivation, and thus damage learning achievement. Additionally, the affective filter hypothesis posits that the emotional states of second-language learners can influence the processing efficiency of language input (Krashen, 1982). Positive emotions such as enjoyment diminish the “Affective Filter,” facilitating the absorption and internalization of language input, thereby improving language proficiency; in contrast, negative emotions such as anxiety and boredom strengthen this filter, hindering language acquisition (Zhou and Wang, 2024). The present study focuses on three types of foreign language learning emotions, among which the positive emotion is denoted as FLE, and the negative emotions are FLA and FLB.

#### 2.3.1 Foreign language anxiety (FLA)

FLA refers to a unique psychological state of learners when learning a foreign language, including nervousness, tension, and fear, which can weaken learners’ confidence and motivation, ultimately failing language development (Horwitz, 2017). Unlike general anxiety, FLA is context-specific and is activated only in situations involving language learning or use (Dewaele et al., 2023). FLA can be affected by learning environments and pedagogical methods, making its mitigation a critical goal for enhancing language learning efficacy (Yu, 2024). It can be seen that FLA is not only a psychological challenge but also a critical variable that influences the effectiveness of language teaching and learning. This study defines FLA as emotional experiences Thai students encounter when learning and speaking Chinese, such as tension and apprehension. In CVT, FLA is considered a “negative activating emotion.” CVT regards FLA as “an emotion connected to learning outcomes” and highlights its disruptive impact on cognitive processes (e.g., distraction, memory inhibition), which could reduce learning performance (Li and Xing, 2024). Liu et al. (2023) noted that personality traits akin to these GR may diminish the generation of negative emotions, such as FLA, by reinforcing a sense of control and value. Educators need to reduce FLA by fostering supportive classroom settings, alleviating assessment pressures, and increasing opportunities for interaction to enhance learning (Dewaele and Macintyre, 2014). COR theory views anxiety as an individual’s stress reaction to the perceived danger or actual deprivation of resources (Hobfoll, 2001). Based on COR theory, FLA refers to the stress experienced by language learners who perceive losing their resources due to insufficient competence, task difficulty, or social pressure. Language learners with adequate resources (e.g., high POE and COI) will transform this anxiety into learning motivation, while those with insufficient resources may experience a decline in performance (Liu et al., 2025).

FLA has garnered heightened interest from scholars during the last two decades. FLA-related studies have been widely undertaken with learners from diverse language backgrounds, proficiency levels, and learning stages. Horwitz (2017) reported that a moderate negative correlation between FLA and language success is prevalent across various languages, regions, and cultures. It is important to acknowledge that Horwitz’s (2017) study did not include any empirical research within the particular setting of CFL. Additionally, correlational research of this nature does not inherently establish that FLA significantly influences language learning achievement. Dewaele et al. (2023) reported that FLA of Moroccan EFL learners significantly affects foreign language achievement, with an impact value that exceeds FLB and FLE. Liu et al. (2025) found that the influences of FLA on language learning achievement are characterized by group-specificity. FLA significantly influenced language learning achievement in the “great effort and interest” profile, whereas no significant effects were observed in other profiles. Li and Wei (2023) discovered that FLA negatively impacted the English performance of EFL learners in rural China in the short term; however, this effect lessened with time. Similarly, Li and Xing (2024) indicated that a moderate level of FLA among Chinese secondary school EFL learners would significantly impact their English learning outcomes. As we can see, research on the influence of FLA on language learning achievements has been thoroughly undertaken within the EFL setting. Compared with English language learning, CFL learners may face unique anxiety due to language features such as Chinese characters and tones, owing to the inherent difficulties related to the Chinese language (Yao et al., 2022). Empirical research on FLA within the context of CFL is limited, and the influence of FLA on student achievement in this domain requires additional investigation.

Yao et al. (2022) conducted systematic review studies on FLA among CFL learners from 1999 to 2020, revealing a significant negative relationship between FLA and performance in Chinese language achievement, particularly exam scores. Most of the studies included in Yao et al.’s (2022) systematic review focused primarily on correlation analyses between FLA and CLA in the context of CFL rather than unilaterally exploring the effects of FLA on CLA. Xu et al. (2022) found that FLA was generally high in Thai adult CFL learners and could significantly negatively affect their CLA. This study used the HSK mock exam to evaluate CLA, contrasting with Xu et al.’s (2022) research, which utilized a hybrid method of subjective self-assessment and vocabulary tests; such differences in measuring instruments may result in divergent research outcomes. Besides, it is noteworthy that in the study by Zhao et al. (2024) on Arab CFL learners, FLA could not influence CLA significantly. Nonetheless, Thai high school students who engage passively in compulsory Chinese courses may markedly differ from the population in Zhao et al.’s (2024) CFL research, which mainly included adult voluntary learners. This study contributes to uncovering challenges faced by Thai students, particularly non-Chinese-major learners without strong self-driven motivation, in the context of ongoing Chinese language education policy implementation. In Thailand’s specific educational context, the sources and mechanisms of FLA among Thai high school students may differ significantly from those of voluntary learners in previous studies and should be further examined. Therefore, the following hypotheses were proposed.

*H3*: FLA is negatively associated with CLA.

#### 2.3.2 Foreign language boredom (FLB)

FLB is recognized as a negative emotion that learners experience in foreign language learning due to poor task design or lack of perceived task value (Li et al., 2023). FLB is closely associated with learner distraction, feelings of underchallenge, decreased motivation, and learning avoidance, all of which impede the development of learners’ language abilities (Li et al., 2024). FLB leads to dissatisfaction, lack of motivation, and attention deficit among foreign language learners, affecting their learning outcomes (Ekatushabe et al., 2021). This research defines FLB as an emotional condition in which Thai students experience boredom with Chinese language learning, resulting in a diminished motivation to engage in the Chinese language classroom. According to the CVT, FLB belongs to a kind of “deactivating emotion,” which causes learners to reduce the allocation of cognitive resources, avoid challenging learning tasks, and ultimately have a negative impact on language learning (He et al., 2025). CVT proposes that an individual’s perception of control and value over achievement tasks and outcomes leads to boredom (Pekrun, 2006). Boredom arises when perceived control is too high or too low and perceived value is insufficient (Goetz et al., 2024). When students encounter boredom in the learning process, they lose their perspective on time, experience exhaustion or frustration, and disengage from their educational efforts (Liu et al., 2025).

Over the past 5 years, FLB has emerged as a focal construct in the burgeoning field of foreign language emotions research. Dewaele et al. (2023) found that Moroccan EFL learners’ FLB had a significant negative effect on their English achievement and stated that it was the second most important emotional factor after FLA. Li (2022) and Zhao and Wang (2023b) found that the FLB of Chinese EFL learners can significantly and negatively affect their English achievement. Li and Wei (2023) longitudinal study on rural Chinese junior high school students revealed that the significant effect of FLB on English performance was found to be short-term and fragile, lasting just 1 month and easily overshadowed by other emotions in the joint model. Notably, despite the substantial body of research on FLB in EFL contexts, most studies have been conducted in China. Research on language learners in other countries and other languages is still severely inadequate. Furthermore, the participants in the aforementioned studies comprised either European adults who actively pursued English or Chinese students who studied English as part of their examination requirements (college entrance or university English examinations), which markedly contrasts with the passive learning tendencies observed among CFL learners in the Thai context. Many Thai students have started learning Chinese since primary school, resulting in a diminished sense of novelty regarding Chinese learning and instruction (Ning and Tananuraksakul, 2024).

In the context of CFL, there exists a scarcity of studies investigating the relationship between FLB and CLA. Pretty (2019) noticed that the FLB experienced by EFL learners at the Confucius Institute at the University of Zimbabwe mainly arises from monotonous teaching methods, which diminish their learning motivation and achievement for studying the Chinese language. However, the study relies solely on qualitative interviews to derive its conclusions, as it lacks quantitative data support, thus potentially limiting its generalizability. Li (2024) stated that in CFL instruction, FLB serves as a critical emotional barrier, indirectly eroding CLA by disrupting the cognitive and motivational processes essential for effective Chinese language acquisition. However, this research concentrates on online CFL education, and the influence of students’ FLB on academic achievement in non-online CFL settings remains unexamined. Also, it is essential to investigate in further detail if FLB directly influences CLA within the CFL context. One of the reasons contributing to the inadequate Chinese language proficiency among many Thai students until the undergraduate level is their lack of enthusiasm for learning Chinese (Xu et al., 2022), which could influence their perception of FLB. Due to the complexity of Chinese and the lack of engaging pedagogical practices in some classrooms, Thai students may develop adaptive strategies toward mandatory Chinese courses, such as reduced engagement, thereby exacerbating FLB (Cao and Tananuraksakul, 2023; Ning and Tananuraksakul, 2024). Based on previous research findings, an in-depth analysis of how FLB influences CLA in the particular context of Thailand is necessary. The researchers proposed the following hypotheses.

*H4*: FLB is positively associated with CLA.

#### 2.3.3 Foreign language enjoyment (FLE)

MacIntyre (2016) defined FLE as learners’ positive emotions associated with completing learning activities, expanding their knowledge, and attaining competency. When learners perceive language learning as inherently enjoyable, their intrinsic motivation escalates, resulting in heightened engagement and enhanced learning achievements (Zeng, 2021). Students who enjoy language learning exhibit less anxiety and heightened motivation to communicate in the target language (Botes et al., 2022). This virtuous loop will enhance learners’ confidence in their language capabilities, hence enhancing language competence (Zhang et al., 2024). The role of FLE is to serve as a motivational “buoy” that assists learners in resisting external pressures and maintaining long-term dedication to learning via dynamic interaction with motivation (Dewaele et al., 2023). This study defines FLE as the emotions of pleasure, vigor, and satisfaction that Thai students experience when learning Chinese. In CVT, FLE is classified as “positive activating emotions,” including feelings of pleasure, happiness, curiosity, and engagement in the learning process (Dewaele and MacIntyre, 2016). As a positive emotion, FLE can boost cognitive resources (e.g., concentration and creativity), hence facilitating active learning among language learners and thereby indirectly improving academic achievements (MacIntyre and Gregersen, 2012). Based on CVT, students who enjoy the foreign language learning process are more likely to be motivated to participate in classroom activities and increase their commitment to learning, which enhances foreign language achievement (Li and Xing, 2024).

Most research related to FLE is undertaken within the context of EFL. Li and Wei’s (2023) longitudinal study found FLE among rural Chinese junior high school EFL learners to be an independent and persistent predictor of English achievement. The studies by Dewaele et al. (2023) on Moroccan EFL learners and Fathi and Hejazi (2024) on Iranian EFL learners consistently conclude that FLE positively and significantly influences their English language achievement. Hu et al. (2024) pointed out that the FLE of Chinese undergraduate-level EFL learners significantly affected English performance. The above studies clearly illustrate the significance of FLE for English language learning achievement. However, substantial distinctions exist between learning Chinese and English, with Chinese often seen as more challenging for non-native speakers (Shen, 2005). The memorization of Chinese characters, mastery of tonal pronunciation, and understanding of culturally loaded vocabulary pose substantial obstacles for CFL learners, resulting in a lack of enjoyment in the Chinese learning process and classroom experiences (Hao, 2012; Yang, 2018).

The impact of FLE on CLA in the context of CFL remains inadequately researched. Zhang and Tsung (2021) investigated international students in Chinese universities and discovered that their FLE could significantly impact their CLA. However, most of these international students are adult learners who voluntarily come to China for their education and are enthusiastic about learning Chinese. Furthermore, they had a more advantageous Chinese language immersion learning environment in China. Hence, the results from Zhang and Tsung (2021) may not be applicable to the present study. Zheng et al. (2023) indicated that FLE is critical to enhancing the learning experience of CFL learners by enhancing their learning motivation and engagement, which in turn influences their CLA. However, this conclusion heavily relies on the interview, and whether FLE significantly affects CLA still needs to be confirmed by empirical research. Thailand is a Buddhist country, and Thai students are influenced by the concepts of “sati” (positive mindfulness) and “sukha” (happiness and fulfillment) and believe that education should be pleasant (Yang and Chanyoo, 2022). In such a cultural context, Thai students will place great importance on the enjoyment of their language learning process. The pronunciation and character writing of Chinese provide considerable difficulties for Thai students, and if they fail to perceive the learning process as sufficiently engaging and enjoyable, their motivation to study further reduces (Ning and Tananuraksakul, 2024). In other words, because Thai students highly value enjoyment, promoting FLE in Chinese classes can reduce learning stress and boost students’ internal motivation to overcome challenges, thus improving Chinese learning outcomes. However, as of now, there is not enough research on FLE for Thai students. Based on previous research findings, the following hypotheses were proposed.

*H5*: FLE is positively associated with CLA.

## 3 Research methods

### 3.1 Research design and sampling

This study focused on high school students studying Chinese at two private and three public schools in Bangkok, Thailand. Employing a quantitative cross-sectional approach, the researchers utilized a questionnaire and the HSK2 mock exam as the research instruments. The convenience sampling method was utilized for this study. Notably, the third author, affiliated with a reputable Chinese language education organization responsible for deploying instructors to various schools in Bangkok, facilitated data collection from a sample comprising 665 participants drawn from 23 classes spanning five schools. The data collection process involved distributing and collecting questionnaires by the six Chinese language teachers affiliated with the educational institution. They also compiled and provided students’ HSK2 scores that were utilized in the analysis.

Participants did not include Chinese nationals, ethnic Chinese who use Chinese at home, or Chinese-major students. The HSK mock exam and questionnaire collection were all conducted in the Chinese classroom, with paper printouts for the HSK exam and Google Forms for the questionnaire. The HSK exam served the primary purpose of assessing the participants’ authentic Chinese proficiency, as they were unaware of the HSK mock exam prior to the lesson and thus had not made adequate preparations for it. This mock examination allocates 50 min for completion, with an additional 10-min interval provided subsequent to its conclusion for students to fill out the questionnaire.

In order to protect the privacy of participants, information such as names or student IDs was not collected from participants. Explicit informed consent was obtained from all participants and their respective guardians to utilize their questionnaire responses and HSK2 scores in the data analysis phase. The researchers ensured the confidentiality and privacy of all participants by deleting demographic information and HSK2 scores within 7 days following the study’s completion. The researchers provided all participants with a small gift of appreciation, valued at approximately 10 baht, as a gesture of gratitude.

Among 665 participants, 228 (34.29%) were males, while 437 (65.71%) were females. Regarding their grade, 326 participants (49.02%) were high school freshmen, 230 participants (34.59%) were high school sophomores, and 109 participants (16.39%) were high school juniors. Moreover, 465 participants (69.92%) studied at public schools, while 200 participants (30.08%) studied at private schools. Regarding their Chinese learning experience, 8 participants (1.20%) had less than 1 year of experience in learning Chinese, 58 participants (8.72%) had between 1 and 3 years of experience, 225 participants (33.83%) had between 3 and 5 years of experience, and 374 participants (56.24%) had more than 5 years of experience.

### 3.2 Research instruments

This study comprises two research instruments: an HSK2 exam paper and a questionnaire.

Following in-depth discussions with several Chinese language teachers in Thailand, the researchers selected the HSK Level 2 exam as the instrument for measuring CLA. The unannounced format prevented last-minute cramming and thus more accurately reflected students’ true Chinese proficiency. Additionally, excluding students aiming to take the Chinese language as an option in Thai university entrance exams or those preparing for HSK to study at Chinese universities, the majority of students do not invest significant time in preparing for Chinese language tests (especially official HSK exams) during their high school years, instead viewing Chinese as a means to explore Chinese culture. Furthermore, most Thai students’ Chinese language proficiency remains inadequate, even at the adult level (Xu et al., 2022). Therefore, at the suggestion of the expert teachers, the researchers uniformly used a beginner-level HSK exam (level 2) to test the CLA of Thai high school students. The HSK exam paper selected for this study is the HSK mock exam sample (H21005), which is available for free on the official website of Chinese Testing International. This mock exam paper has two sections: Listening and Reading. It consists of 60 questions, with a combined score of 200 points (100 points for each section). A score of 120 points or above (60%) is the threshold for a passing grade.

The questionnaire consisted of two sections: demographic questions and scales. The researchers collected participants’ demographic information, including age, gender, school, and Chinese learning experiences, but not names or student IDs. All scales were on a five-point Likert scale (1 = Strongly Disagree, 5 = Strongly Agree). Before the formal distribution of the questionnaires, the researchers conducted a pre-test by distributing 50 questionnaires to students in two Bangkok high schools (one private and one public) to establish whether the selected scales were internally consistent and reliable.

The Child and Adolescent Social Support Scale (CASSS), developed by Malecki and Demaray (2002), systematically measures social support from parents, teachers, classmates, and friends during the learning process, and the PTS scale for the present study adapted 10 items from the CASSS regarding teacher support. In the pre-test, one of the items, “My Chinese teacher helps me when I get confused,” was removed by researchers because its Corrected Item-Total Correlation (CITC) value was too low (0.298). The remaining item had a Cronbach’s alpha of 0.728, which has sufficient internal consistency. The GR scale was adapted from Teimouri et al. (2022), with two sub-dimensions and nine items, of which persistence of effort (POE) consists of five items and consistency of interest (COI) consists of four items. In the pre-test, the Cronbach’s alpha for the two sub-dimensions was 0.852 and 0.880, so this scale had sufficient internal consistency. The FLE scale was adapted from the nine-item Short Form of the Foreign Language Enjoyment Scale (S-FLES) developed by Botes et al. (2021), which consists of three sub-dimensions: Teacher Appreciation (TA), Personal Enjoyment (PE), and Social Enjoyment (SE). In the pre-test, the item from SE, “We have common ‘legends,’ such as running jokes,” was removed because it had a low CITC value (0.354). After excluding this item, the Cronbach’s alphas for the three sub-dimensions in this study were 0.820, 0.788, and 0.724, with sufficient internal consistency. The Foreign Language Classroom Anxiety Scale (FLCAS) is the classic instrument most commonly used to measure FLA (Horwitz, 2017). The FLA scale in this study utilized Dewaele and Macintyre (2014) 8-item Short Form of the Foreign Language Classroom Anxiety Scale (S-FLCAS), which is more brief but still has sufficient validity. In the pre-test, this scale had a Cronbach’s alpha of 0.824. The FLB scale, adapted from the 8-item Foreign Language Boredom Scale (FLBS) developed by Li et al. (2023), is used to measure boredom levels among Thai students in Chinese language classrooms. This scale had a Cronbach’s alpha of 0.902 in the pre-test.

### 3.3 Data analysis

**Figure 1:**
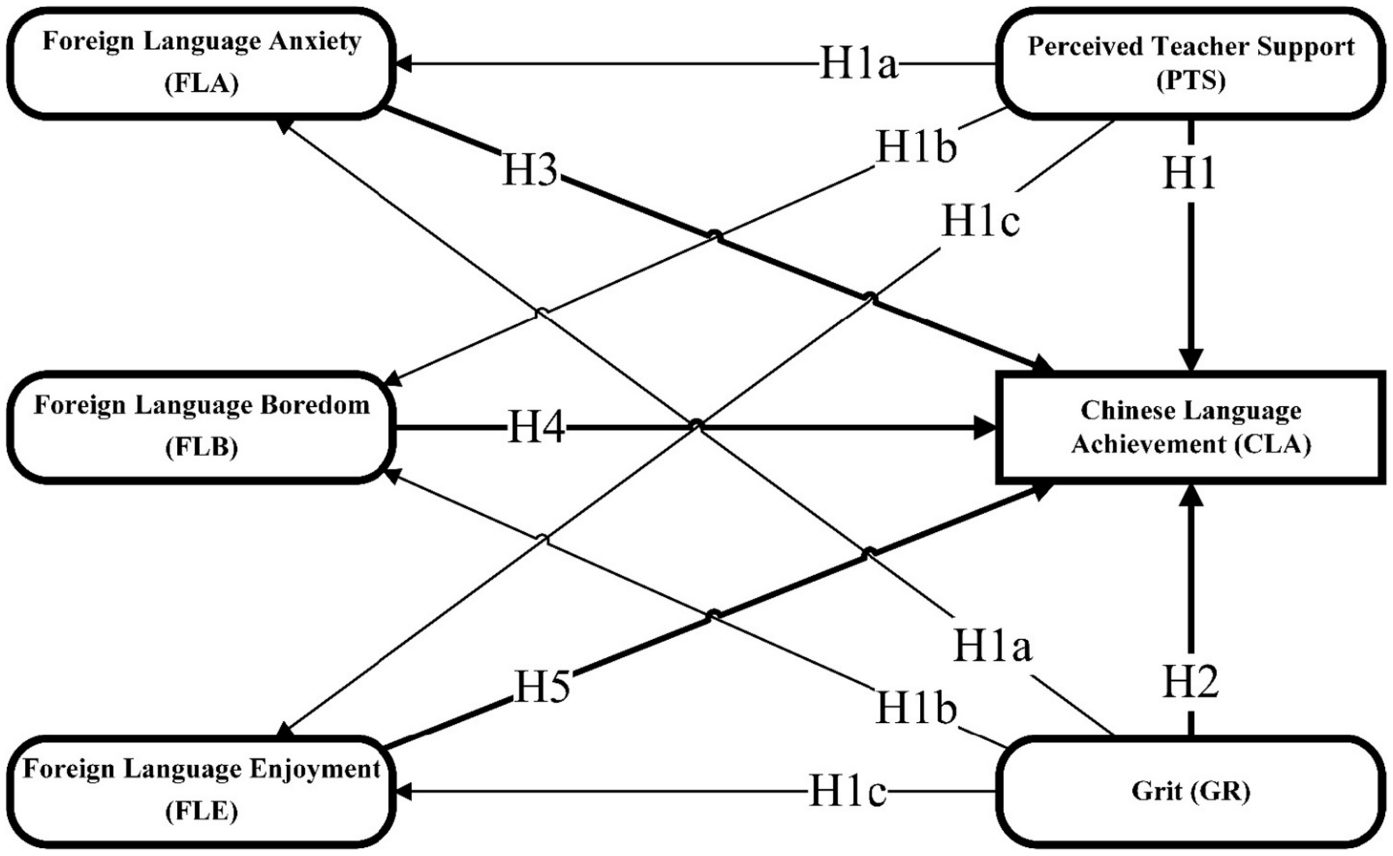
The conceptual framework.

First, the researchers utilized SPSS Statistics 24 software to analyze the frequencies and percentages of all demographic information. After that, through SmartPLS 4 software, the researchers conducted step-by-step analyses of internal consistency (reliability) and convergent and discriminant validity for all constructs (scales) in this study. After re-establishing that the proposed model has sufficient reliability and validity, the researchers proceeded to hypothesis testing. Although the sample size of this study was quite sufficient, the researchers still chose Partial Least Squares Structural Equation Modelling (PLS-SEM), not Covariance-based Structural Equation Modelling (CB-SEM). PLS-SEM was chosen because the proposed model simultaneously considered PTS, GR, and emotional factors to develop an innovative conceptual framework for predicting Thai students’ CLA. PLS-SEM is more suitable for testing exploratory models, while CB-SEM is more suitable for re-testing classical theoretical models (Afthanorhan et al., 2020). Besides, both GR and FLE scales in this study contain sub-dimensions; thus, the model is higher-order. PLS-SEM is relatively more convenient and suitable for predicting higher-order models than CB-SEM (Hair et al., 2013). Finally, the analysis of indirect effects was also performed with the SmartPLS 4 software (Figure 1).

## 4 Results

### 4.1 Descriptive analysis

Table 1 presents the basic information of the descriptive statistics for all constructs, covering the maximum value, minimum value, mean, standard deviation, skewness, and kurtosis. It can be observed that the mean values of the two variables (SE and TA) within FLE are relatively high (greater than 3.5), while the mean values of the two variables (COI and POE) in Grit are relatively low (less than 2.5). The mean values of the remaining variables are at moderate levels. The mean value of the students’ HSK scores (CLA) is 118.91, with a standard deviation of 32.72.

**Table 1:** Descriptive analysis.

Variables	Min	Max	Mean	Std. Deviation	Skewness	Kurtosis
FLA	1.25	4.63	2.89	0.67	−0.038	−0.373
FLB	1.00	4.75	2.99	0.69	−0.142	−0.597
PE	1.33	5.00	3.02	0.65	−0.024	−0.492
SE	1.50	5.00	3.55	0.68	0.021	−0.239
TA	1.33	5.00	3.59	0.66	−0.022	0.153
COI	1.25	5.00	2.46	0.47	−0.034	−0.532
POE	1.40	4.60	2.43	0.50	−0.067	−0.201
PTS	1.50	5.00	3.39	0.59	−0.083	−0.261
HSK	65.00	200.00	118.91	32.72	0.774	−0.088

**Figure 2:**
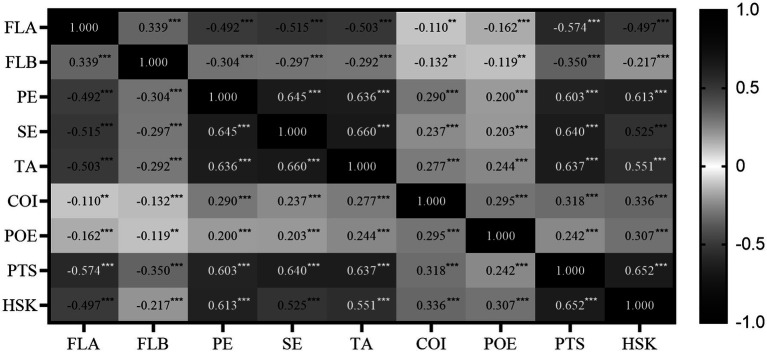
Heat map (correlation matrix).

When skewness and kurtosis approach zero, the data are considered to follow a normal distribution. In PLS-SEM analysis, when skewness and kurtosis are within the range of ±2, the data can be regarded as approximately normally distributed (Hair and Alamer, 2022). As shown in Table 1, the skewness and kurtosis of each variable fall within the range of ±2. Therefore, all these variables tend to follow a normal distribution. Since all variables tend to follow a normal distribution, the researchers employed the Pearson correlation coefficient matrix to measure the relationship among variables. As depicted in Figure 2, significant correlations exist among all variables. Notably, the two variables, FLA and FLB, exhibit correlations in different directions with other variables.

### 4.2 Measurement model

The researchers first analyzed the presence of multicollinearity for individual items in the model. The Variance Inflation Factor (VIF) is an important indicator used to measure multicollinearity, and generally, a VIF value below 5 proves that there is no severe multicollinearity problem (Kim, 2019). The VIF values in this study ranged from 1.753 to 2.789, meaning there is no severe issue of multicollinearity.

Cronbach’s alpha was used to evaluate the internal consistency of items within each dimension. A Cronbach’s alpha above 0.7 indicated both internal consistency throughout dimensions and the reliability of the study. As shown in Table 2, Cronbach’s alpha for the constructs (scales) ranged from 0.770 to 0.930, showing good internal consistency and reliability.

**Table 2:** Reliability and convergent validity.

Construct	Items	Factor loading	Alpha	CR	AVE
GR—POE	POE1	0.763	0.848	0.848	0.622
	POE2	0.781			
	POE3	0.790			
	POE4	0.819			
	POE5	0.787			
GR—COI	COI1	0.770	0.814	0.816	0.642
	COI2	0.814			
	COI3	0.819			
	COI4	0.802			
PTS	PTS1	0.766	0.899	0.899	0.585
	PTS2	0.760			
	PTS3	0.803			
	PTS4	0.759			
	PTS5	0.756			
	PTS6	0.797			
	PTS7	0.738			
	PTS8	0.737			
FLA	FLA1	0.787	0.927	0.927	0.661
	FLA2	0.813			
	FLA3	0.845			
	FLA4	0.797			
	FLA5	0.825			
	FLA6	0.836			
	FLA7	0.809			
	FLA8	0.792			
FLE—PE	PE1	0.876	0.845	0.845	0.763
	PE2	0.876			
	PE3	0.868			
FLE—TA	TA1	0.870	0.837	0.838	0.754
	TA2	0.876			
	TA3	0.860			
FLE—SE	SE1	0.898	0.770	0.771	0.813
	SE2	0.906			
FLB	FLB1	0.842	0.930	0.933	0.661
	FLB2	0.809			
	FLB3	0.771			
	FLB4	0.803			
	FLB5	0.849			
	FLB6	0.821			
	FLB7	0.853			
	FLB8	0.810			

Convergent validity can be assessed by examining factor loading, composite reliability, and average variance extracted (AVE). Factor loadings reflect the correlation between observed and latent variables, with values above 0.6 deemed acceptable (Awang, 2015). The factor loadings of the scale items in this study are shown in Table 2 and ranged from 0.737 to 0.906, which is within the acceptable range. Composite reliability (CR) measured the model’s internal consistency, with a threshold of 0.7 regarded as acceptable (Hair, 2010). As shown in Table 2, the CRs in this study ranged from 0.770 to 0.933, demonstrating that all constructs (scales) had sufficient internal consistency. The Average Variance Extracted (AVE) serves as a crucial metric for evaluating convergent validity, with an AVE value exceeding 0.5 denoting satisfactory convergent validity (Fornell and Larcker, 1981). As shown in Table 2, the AVE values for all constructs (scales) ranged from 0.585 to 0.813, which are all within the acceptable range. Therefore, all constructs in this study have sufficient convergent validity.

Discriminant validity can be established when the square root of the AVE for each dimension exceeds the correlation coefficients of the other dimensions associated with it (Fornell and Larcker, 1981). As shown in Table 3, the square root of the AVE values for all constructs (scales) in this study was greater than the corresponding correlation coefficients. Therefore, the present study has sufficient discriminant validity. The heterotrait-monotrait ratio of correlations (HTMT) is a novel measure of whether latent variables have discriminant validity (Henseler et al., 2015). When all values in the HTMT matrix are less than 0.85, the study has sufficient discriminant validity. As shown in Table 4, the HTMT matrix shows that the HTMT values between all the constructs (scales) were less than 0.85, and the discriminant validity of the study was again validated.

**Table 3:** Fornell-Larcker criterion.

Variables	FLA	FLB	PE	SE	TA	CI	POE	PTS
FLA	**0.813**							
FLB	0.339	**0.820**						
PE	−0.492	−0.304	**0.873**					
SE	−0.515	−0.297	0.645	**0.902**				
TA	−0.503	−0.293	0.636	0.660	**0.869**			
CI	−0.110	−0.133	0.291	0.237	0.277	**0.801**		
POE	−0.162	−0.119	0.201	0.203	0.244	0.295	**0.788**	
PTS	−0.574	−0.353	0.604	0.640	0.637	0.318	0.242	**0.765**

The bold values represent the square roots of the average variance extracted (AVE).

**Table 4:** Heterotrait-monotrait ratio of correlations (HTMT).

Variables	FLA	FLB	PE	SE	TA	CI	POE	PTS
FLA								
FLB	0.364							
PE	0.556	0.341						
SE	0.609	0.349	0.799					
TA	0.570	0.331	0.756	0.821				
CI	0.127	0.152	0.350	0.298	0.334			
POE	0.184	0.136	0.237	0.252	0.290	0.353		
PTS	0.629	0.383	0.692	0.768	0.734	0.372	0.278	

### 4.3 Structural model

Once the reliability and validity were established, the researchers proceeded to test the hypotheses using SmartPLS 4.0. The percentile bootstrap technique with 5,000 iterative resamples was used in this research, as suggested by Hair et al. (2017).

**Figure 3:**
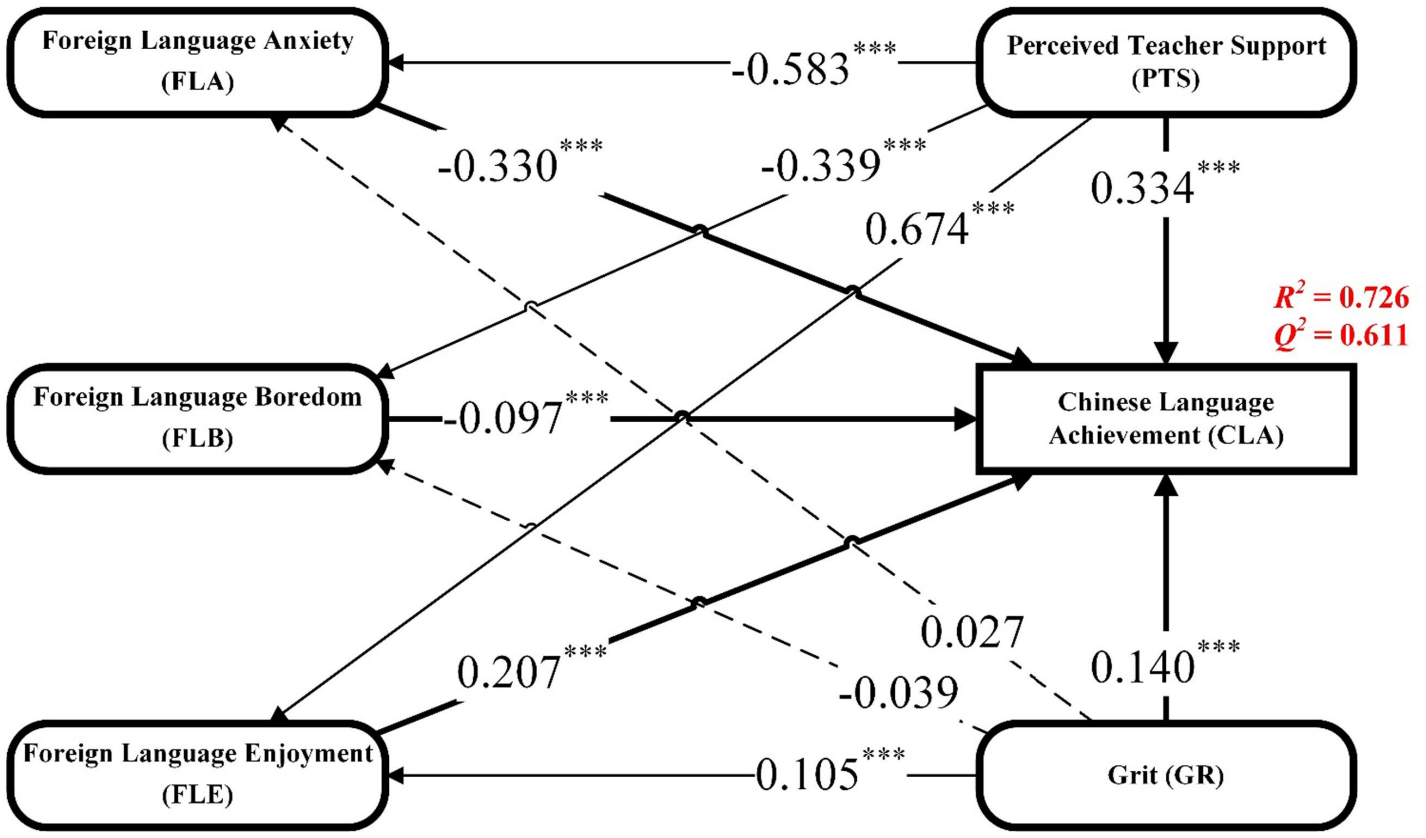
PLS results.

As shown in Table 5, all the constructs were significantly related to CLA. PTS had the strongest significant and positive association with CLA (*β* = 0.334, *p* < 0.001), while FLA (*β* = −0.330, *p* < 0.001) and FLB (*β* = −0.097, *p* < 0.001) were significantly and negatively associated with CLA, and GR (*β* = 0.140, *p* < 0.001) and FLE (*β* = 0.207, *p* < 0.001) were significantly and positively associated with CLA. PTS was significantly associated with all of the emotional factors. In particular, PTS was significantly and positively associated with FLE (*β* = 0.674, *p* < 0.001) and was significantly and negatively associated with FLA (*β* = −0.583, *p* < 0.001) and FLB (*β* = −0.339, *p* < 0.001). GR was not significantly associated with FLB (*β* = −0.039, *p* = 0.315) and FLA (*β* = 0.027, *p* = 0.417), while it was significantly and positively associated with FLE (*β* = 0.105, *p* < 0.001). The results of PLS-SEM in this study are shown in Figure 3.

**Table 5:** Hypothesis testing.

Hypotheses	Path	Original sample (O)	STDEV	*t*	*p*-values	Results
H1	PTS → CLA	0.334	0.029	11.470	***	Supported
H1a	PTS → FLA	−0.583	0.027	21.574	***	Supported
H1b	PTS → FLB	−0.339	0.036	9.462	***	Supported
H1c	PTS → FLE	0.674	0.021	31.741	***	Supported
H2	GR → CLA	0.140	0.022	6.326	***	Supported
H2a	GR → FLA	0.027	0.033	0.811	0.417	Not Supported
H2b	GR → FLB	−0.039	0.039	1.004	0.315	Not Supported
H2c	GR → FLE	0.105	0.029	3.620	***	Supported
H3	FLA → CLA	−0.330	0.025	13.266	***	Supported
H4	FLB → CLA	−0.097	0.021	4.569	***	Supported
H5	FLE → CLA	0.207	0.027	7.596	***	Supported

As shown in Table 6, FLA (*β* = 0.192, *p* < 0.001), FLB (*β* = 0.033, *p* < 0.001), and FLE (*β* = 0.140, *p* < 0.001) were each significant mediators of the association between PTS and CLA; FLA (*β* = 0.009, *p* = 0.420) and FLB (*β* = 0.004, *p* = 0.333) did not mediate the association between GR and CLA, while FLE (*β* = 0.022, *p* = 0.001) did.

**Table 6:** Indirect effects.

Path	Original sample (O)	STDEV	*t*	*p*-values
PTS → FLA → CLA	0.192	0.017	11.316	***
PTS → FLB → CLA	0.033	0.008	4.041	***
PTS → FLE → CLA	0.140	0.019	7.348	***
GR → FLA → CLA	0.009	0.011	0.807	0.420
GR → FLB → CLA	0.004	0.004	0.968	0.333
GR → FLE → CLA	0.022	0.007	3.289	0.001**

The coefficient of determination (*R^2^*) was employed to measure the proportion of the dependent variable that was predicted by the independent variable (Cohen, 2013). In this study, the *R^2^* for CLA was 0.726, which means that GR, PTS, and emotional factors explain 72.6% of Chinese learning achievement. The predictive relevance of a model is evaluated using *Q^2^*, whereby a value greater than 0 indicates the model’s satisfactory predictive capability. The *Q^2^* of Chinese language learning is 0.611, which means the model has a good predictive ability for Chinese language learning. F-squared (*f^2^*) denotes the change in *R^2^* when an exogenous variable is excluded from the model. According to Cohen (2013), an effect size is considered small when *f^2^* is higher than 0.02, medium when *f^2^* is higher than 0.15, and large when *f^2^* is higher than 0.35. In the current study, FLA (*f^2^* = 0.237) and PTS (*f^2^* = 0.178) had moderate effects on CLA, and GR (*f^2^* = 0.062) and FLE (*f^2^* = 0.069) had small effects on CLA.

## 5 Discussion and implications

Although research on language achievement has long been a popular topic in studies related to foreign language learning, most research has been conducted in EFL contexts, and there is a serious shortage of research on CFL. This study aimed to examine the associations of GR, PTS, and emotions (FLA, FLB, FLE) with CLA in Chinese language learning among Thai high school students. By considering both the associations of student factors (GR) and teacher factors (PTS) with CLA, as well as the mediating role of emotional factors in CLA, the proposed model has good explanatory power (*R^2^* = 0.726) for Thai students’ CLA. Thus, it offers a novel perspective and a multi-dimensional model for future research on CFL and other foreign language learning contexts. Besides, this study elucidated the influence of “positive mindfulness” and “happiness and fulfillment” on students’ emotional experiences and learning behaviors within Thai Buddhist culture, offering a novel interpretation of cross-cultural language learning theory, which posits that cultural values enhance motivation for language acquisition and subsequently improve learning achievement. Future research could elevate cultural values from ‘background variables’ into ‘explanatory variables,’ thereby enriching the explanatory framework of cross-cultural linguistic studies. A detailed discussion of the results is described below.

According to the findings, PTS is the strongest predictor of CLA, and it is positively and significantly associated with CLA (*H1*), consistent with previous studies’ results (Hejazi and Sadoughi, 2023; Tao et al., 2022). By providing various kinds of assistance, assessment, and feedback based on learners’ abilities, teachers can contribute to students’ success (Leon et al., 2017). Many Thai students encounter difficulties and challenges in learning Chinese, and the support provided by Chinese teachers motivates them to keep learning Chinese, which also benefits their language achievement. Regarding the association between PTS and emotions, firstly, Thai students’ PTS was negatively and significantly associated with FLA (*H1a*), consistent with previous studies’ findings (Liu et al., 2023; Piechurska-Kuciel, 2011). Providing sufficient support by CFL teachers can alleviate anxiety among Thai students in the Chinese classroom, fostering a more relaxed learning environment. Likewise, PTS was negatively and significantly associated with FLB (*H1b*), which is consistent with the results of previous studies (Wu et al., 2023; Zhao and Yang, 2022). As Kruk and Zawodniak (2018) pointed out, teachers are the most significant contributors to student boredom. When students recognize that CFL teachers want to provide them with support, their psychological needs are fulfilled, hence enhancing cognitive engagement and emotional empathy, which mitigates their boredom in the learning process. Furthermore, PTS was also positively and significantly associated with FLE (*H1c*), consistent with some previous studies (Liu and Zhou, 2024; Zhao and Yang, 2022). When Thai students perceive adequate support from their CFL teachers, harmonious teacher-student relationships and classroom environments are fostered, enhancing their enjoyment of Chinese language learning. The strong associations of PTS with three key emotions also highlight the importance of teacher support in language learning.

This study also found that all three language-learning emotions (FLA, FLB, and FLE) were able to mediate the associations between PTS and CLA significantly. First, FLA served as a mediator for the relationship between PTS and CLA. Thai students’ FLA tends to decline when they believe their CFL teachers provide sufficient support (such as timely error correction or interesting learning materials), reducing cognitive load and enhancing CLA. FLB can also mediate the association between PTS and CLA. Students feel less bored when they believe that their teachers are supporting them, for example, by implementing gamified teaching or a variety of instructional activities like role-playing in the classroom. This decrease in boredom, in turn, improves focus and indirectly leads to better academic achievement. Students’ interest and curiosity about language learning are piqued when they believe that their CFL teachers offer assistance, such as supportive feedback and autonomy support. Consequently, this leads to improved language proficiency. CFL teachers should take the initiative to get to know their students and give customized support based on their language level and emotional needs, making more students feel that their teachers are offering them support. Such personalized teaching strategies could potentially reduce learning-related anxiety and boredom, foster enjoyment in Chinese language learning, and ultimately improve long-term language outcomes.

Many CFL teachers, especially inexperienced ones, often struggle to ensure their support is effectively perceived by students when providing assistance. Some CFL teachers in Thailand continue to encounter many challenges, including language communication barriers and insufficient cultural acclimatization (Duangmanee and Waluyo, 2023). Language barriers could hinder trust-building between teachers and students, while cultural misunderstandings limit CFL teachers’ local adaptation of teaching methods (Wang and Du, 2016). These challenges undermine Thai students’ PTS and hinder the development of language proficiency. CFL teachers should systematically improve intercultural competence and local language proficiency through customized language training or cultural immersion (e.g., participation in intensive Thai language courses and Buddhist cultural studies). Also, CFL teachers can provide students with interesting insights about Chinese culture while simultaneously encouraging them to share their knowledge of Thai culture in the Chinese language classroom, thus fostering cultural exchange and mutual understanding. Thai schools and educational institutions can frequently organize cross-cultural communication activities between CFL teachers and local teachers to enhance CFL teachers’ local cultural interpretation capabilities and instructional strategy adaptability while fostering mutual collaboration among educators. Educational administrators evaluating CFL teachers should not solely focus on teachers’ Chinese proficiency and instructional methodologies but prioritize student perspectives and evaluations, given that students’ perceived teacher support significantly contributes to enhancing their language learning emotions and outcomes.

Thai students’ GR was significantly and positively associated with their CLA (*H2*), consistent with extensive previous research (Fathi and Hejazi, 2024; Teimouri et al., 2022). Chinese language learning is challenging, and learners must spend much time and energy on it (Steinmayr et al., 2018). The GR exhibited by Thai students, particularly their perseverance in Chinese language learning over the long term, plays a vital role in their capacity to persist through the entire learning process, subsequently influencing their CLA. Furthermore, Thai students’ GR was significantly and positively associated with their FLE (*H2a*), consistent with the findings of previous studies (Bensalem et al., 2024; Khajavy and Aghaee, 2024). Learners who maintain consistent engagement in language learning over an extended period tend to report higher levels of enjoyment (Yeşilçınar and Erdemir, 2023). CFL learners who possess a high level of GR are more likely to consider Chinese language learning as their interest, and they often watch Chinese movies, read Chinese books, and participate in Chinese cultural activities, which makes them believe Chinese learning is enjoyable (Wang et al., 2022; Zhao and Wang, 2024). Interestingly, it was found that GR was not significantly associated with FLA and FLB (*H2b & H2c*). Although some Thai students possess high GR in Chinese language learning, they may still lack confidence in their proficiency. It can lead to FLA and FLB in the classroom, where students may be afraid of making mistakes or being judged by their teacher. Many CFL learners try to persist in learning Chinese, but they still experience FLA and FLB due to cultural differences, lack of confidence, inappropriate teaching methods, and depressing learning environment (Chen et al., 2024; Tai and Wang, 2024). Furthermore, FLE can mediate the association between GR and CLA, but FLA and FLB cannot. Thus, among Thai CFL learners, GR promotes CLA primarily through fostering learning enjoyment rather than alleviating anxiety and boredom. Thai CFL learners with higher GR may consciously create environments that make learning more engaging, such as selecting interesting materials and implementing self-reward mechanisms, thereby enhancing FLE to enhance CLA. Once again, it revealed the importance of GR for Thai students learning Chinese and the feasibility of influencing FLE and thus increasing CLA by raising the GR of Thai students learning Chinese.

Despite years of study, Thai students’ confidence and GR are undermined by the significant challenges in learning Chinese, which include tonal complexity, character writing, grammatical disparities, and cultural differences. Thai Buddhist culture emphasizes “patience” and “long-term practice,” so Thai students generally have a relatively high level of GR (Freiermuth et al., 2021). CFL teachers should tailor customized learning content for different learners, provide prompt feedback and encouragement, and assist students in overcoming challenges, thus bolstering their GR in learning Chinese. Another effective approach is setting a good example for students by showcasing teachers’ language learning journeys (e.g., learning Thai) while highlighting classmates demonstrating dedication to Chinese studies. This helps students internalize the understanding that Chinese language learning requires sustained effort, fostering the development of GR. Thai schools and educational institutions could frequently organize Chinese language activities, such as Chinese knowledge competitions, Chinese speeches, Chinese cultural exhibitions, etc., to give students opportunities to display their talents, stimulate their motivation, and thus cultivate their grit. Furthermore, it is also a great way to provide Thai students with Chinese learning resources they are interested in, such as Chinese novels, skits, videos, etc., which facilitates students’ independent learning, meets their long-term learning needs, and thus enhances GR.

FLA was significantly and negatively associated with CLA (*H3*), consistent with previous studies (Li and Wei, 2023; Li and Xing, 2024). In the Chinese language classroom, when Thai students feel FLA, they may have difficulty concentrating on instruction, memorizing knowledge, and interacting with teachers, eventually leading to lower Chinese achievement. Therefore, CFL teachers are encouraged to cultivate a safe, friendly, and supportive environment in the classroom for their students, promoting more engagement in classroom activities to alleviate their anxieties and tensions. Some CFL teachers frequently conduct quizzes (e.g., Chinese dictation, random questions, grammar drills, etc.) in class, which can cause anxiety among unprepared students. Reducing the frequency of these quizzes, providing advance notice of upcoming tests, and encouraging students to prepare are highly effective in reducing FLA among Thai students. For Thai schools and educational institutions, providing timely psychological counseling and tutoring services can effectively alleviate Thai students’ FLA and promote mental health, thus enhancing their Chinese learning outcomes.

FLB was significantly and negatively associated with CLA (*H4*), consistent with previous studies (Li, 2022; Zhao and Wang, 2023b). Boredom in the Chinese classroom among Thai students can decrease their engagement in the classroom. In Chinese classrooms, students’ lack of engagement will lead to an inability to concentrate on listening and actively participate in classroom interactions, ultimately affecting students’ CLA. CFL teachers can enhance Thai students’ Chinese learning by breaking down long-term goals into short-term achievable sub-goals and consistently fostering their motivation through incremental progress. Besides, timely encouragement and affirmation of Thai students’ previous CLA are effective strategies to maintain their motivation and enable them to persist in learning Chinese in the long term. Thai schools and educational institutions can optimize Chinese curriculum design by tailoring objectives for non-specialized learners and encouraging CFL teachers to integrate engaging cultural activities like calligraphy, music, and painting into lessons.

Lastly, FLE was positively and significantly associated with CLA (*H5*), consistent with previous studies (Hu and Bentler, 1999; Li and Wei, 2023). When Thai students have a strong FLE of Chinese language learning, they are more likely to be active, participate in classroom activities, and engage in extra practices, ultimately leading to students’ success in Chinese language learning. Therefore, CFL teachers are expected to optimize their teaching methods to make the Chinese language classroom more interesting. For instance, integrating elements of interest to students (such as Chinese movies or games they engage with) into classroom activities can enhance learner engagement and interactivity, fostering an enjoyable and relaxed Chinese learning environment. Thai schools and educational institutions are encouraged to organize Chinese language activities on specific dates (e.g., Chinese New Year and Dragon Boat Festival) so that students can feel the enjoyable atmosphere of Chinese festivals and also learn Chinese while embracing Chinese culture.

## 6 Conclusion

This study aimed to explore the effects of Thai high school-level CFL learners’ emotions (FLA, FLB, and FLE), PTS, and GR on CLA. The mediating effects of the three emotional variables on the relationships between PTS and CLA, as well as between GR and CLA, were also analyzed. Utilizing a cross-sectional survey design, this study involved students from five high schools in Bangkok. The researchers collected 665 valid data through a questionnaire and an HSK2 mock exam, which were analyzed using PLS-SEM. The hypothesis testing findings demonstrated that all PTS, GR, and emotion factors significantly impacted CLA. Besides, PTS was found to have a significant impact on all three emotions, whereas GR had a significant effect only on FLE. All three emotions were found to mediate the effects of PTS on CLA significantly; however, only FLE could mediate the effects of GR on CLA. This study emphasizes the importance of creating a supportive classroom environment, meeting students’ affective needs, and implementing diverse teaching strategies to cultivate Thai students’ long-term learning perseverance and thus improve their Chinese language learning achievement.

## 7 Limitations

The following limitations exist in this study. Firstly, the study employed a cross-sectional design, which limits its ability to establish causal relationships between variables. While the model revealed associations among PTS, GR, emotions, and CLA, it cannot definitively establish causal effects. It is recommended that future research employ longitudinal designs to track the dynamic evolution of these factors over time and their reciprocal influences. Second, as the study’s sample included only CFL learners from Bangkok high schools, its conclusions may not be generalizable to other regions (particularly those outside Buddhist cultural contexts). Therefore, the researchers recommend that future studies employ or adapt this research model when conducting similar research in diverse regions to enhance the external validity. Lastly, this research did not consider the impact of external control variables on its results, and diverse demographic factors could affect the model’s stability and prediction accuracy. Future research is advised to incorporate control variables such as gender, grade, and Chinese learning experience to enhance the model’s predictive accuracy.

## Data Availability

The raw data supporting the conclusions of this article will be made available by the authors without undue reservation.
