# Supplementary material for: Grit and perceived teacher support associations with Chinese language achievement: the mediating role of emotion in Thai high school Chinese classrooms
Source: Front Psychol. 2025 May 26;16:1573713. doi: 10.3389/fpsyg.2025.1573713 (PMC12146286; doi:10.3389/fpsyg.2025.1573713)
Supplement: Supplementary file 1 [file Data_Sheet_1.pdf]

Table 1s. Model Invariance Analysis Across Grades

		**Difference (Grade 1 - Grade 2)**	***p*-value (Grade 1 - Grade 2)**	**Difference (Grade 1 - Grade 3)**	***p*-value (Grade 1 - Grade 3)**	**Difference (Grade 2 - Grade 3)**	***p*-value (Grade 2 - Grade 3)**
H1	**GRIT -> CLA**	0.006	0.909	-0.075	0.236	-0.081	0.138
H1a	**GRIT -> FLA**	-0.060	0.353	-0.089	0.190	-0.029	0.601
H1b	**GRIT -> FLB**	-0.103	0.106	-0.096	0.184	0.007	0.881
H1c	**GRIT -> FLE**	0.004	0.945	-0.042	0.633	-0.046	0.616
H2	**PTS -> CLA**	-0.032	0.620	-0.003	0.971	0.030	0.716
H2a	**PTS -> FLA**	0.054	0.356	0.085	0.222	0.031	0.688
H2b	**PTS -> FLB**	0.020	0.800	0.025	0.791	0.004	0.946
H2c	**PTS -> FLE**	-0.027	0.542	0.023	0.736	0.050	0.452
H3	**FLA -> CLA**	0.027	0.645	0.088	0.223	0.061	0.452
H4	**FLB -> CLA**	-0.067	0.322	-0.043	0.452	0.024	0.376
H5	**FLE -> CLA**	0.016	0.798	0.075	0.269	0.059	0.417

Table 2s. Model Invariance Analysis Across Genders (Female – Male)

**Hypothesis**	**Path**	**Difference**	***p*-value**
H1	GRIT -> CLA	0.034	0.435
H1a	GRIT -> FLA	0.016	0.814
H1b	GRIT -> FLB	-0.052	0.352
H1c	GRIT -> FLE	0.057	0.322
H2	PTS -> CLA	-0.086	0.124
H2a	PTS -> FLA	-0.040	0.448
H2b	PTS -> FLB	0.096	0.144
H2c	PTS -> FLE	-0.005	0.896
H3	FLA -> CLA	0.026	0.591
H4	FLB -> CLA	0.011	0.795
H5	FLE -> CLA	0.071	0.182

Table 3s. Model Invariance Analysis Across Experiences (Less than 5 years – More than 5 years)

**Hypothesis**	**Path**	**Difference**	***p*-value**
H1	GRIT -> CLA	0.049	0.326
H1a	GRIT -> FLA	0.051	0.459
H1b	GRIT -> FLB	0.051	0.421
H1c	GRIT -> FLE	0.026	0.653
H2	PTS -> CLA	0.013	0.826
H2a	PTS -> FLA	0.034	0.548
H2b	PTS -> FLB	0.073	0.327
H2c	PTS -> FLE	-0.020	0.655
H3	FLA -> CLA	-0.029	0.562
H4	FLB -> CLA	0.037	0.471
H5	FLE -> CLA	-0.063	0.259

Table 4s. Model Invariance Analysis Across Schools (Private school – Public school)

**Hypothesis**	**Path**	**Difference**	***p*-value**
H1	GRIT -> CLA	0.021	0.659
H1a	GRIT -> FLA	0.059	0.412
H1b	GRIT -> FLB	-0.046	0.571
H1c	GRIT -> FLE	0.049	0.424
H2	PTS -> CLA	0.031	0.624
H2a	PTS -> FLA	0.038	0.563
H2b	PTS -> FLB	0.104	0.072
H2c	PTS -> FLE	-0.035	0.453
H3	FLA -> CLA	-0.02	0.698
H4	FLB -> CLA	-0.022	0.654
H5	FLE -> CLA	-0.044	0.464
